# Phenotypic and metabolic responses to drought and salinity of four contrasting lentil accessions

**DOI:** 10.1093/jxb/erv208

**Published:** 2015-05-11

**Authors:** A. Muscolo, A. Junker, C. Klukas, K. Weigelt-Fischer, D. Riewe, T. Altmann

**Affiliations:** ^1^Agriculture Department, Mediterranea University, Feo di Vito, 89124 Reggio Calabria, Italy; ^2^Department of Molecular Genetics, Leibniz-Institute of Plant Genetics and Crop Plant Research (IPK) Gatersleben, Corrensstrasse 3, D-06466 Stadt Seeland OT Gatersleben, Germany

**Keywords:** Drought stress, lentil, metabolite profiling, phenotypic traits, salinity, seed germination.

## Abstract

Drought and salinity are among the major abiotic stresses which, often inter-relatedly, adversely affect plant growth and productivity. Plant stress responses depend on the type of stress, on its intensity, on the species, and also on the genotype. Different accessions of a species may have evolved different mechanisms to cope with stress and to complete their life cycles. This study is focused on lentil, an important Mediterranean legume with high quality protein for the human diet. The effects of salinity and drought on germination and early growth of Castelluccio di Norcia (CAST), Pantelleria (PAN), Ustica (UST), and Eston (EST) accessions were evaluated to identify metabolic and phenotypic traits related to drought and/or salinity stress tolerance. The results showed a relationship between imposed stresses and performance of the cultivars. According to germination frequencies, the accession ranking was as follows: NaCl resistant > susceptible, PAN > UST > CAST > EST; polyethylene glycol (PEG) resistant > susceptible, CAST > UST > EST > PAN. Seedling tolerance rankings were: NaCl resistant > susceptible, CAST ≈ UST > PAN ≈ EST; PEG resistant > susceptible, CAST > EST ≈ UST > PAN. Changes in the metabolite profiles, mainly quantitative rather than qualitative, were observed in the same cultivar in respect to the treatments, and among the cultivars under the same treatment. Metabolic differences in the stress tolerance of the different genotypes were related to a reduction in the levels of tricarboxylic acid (TCA) cycle intermediates. The relevant differences, between the most NaCl-tolerant genotype (PAN) and the most sensitive one (EST) were related to the decrease in the threonic acid level. Stress-specific metabolite indicators were also identified: ornithine and asparagine as markers of drought stress and alanine and homoserine as markers of salinity stress.

## Highlight

Automated imaging-based plant phenotyping combined with GC-MS-based metabolite profiling of four lentil accessions differing in their drought and salt tolerance shows common and specific responses and yields characteristic stress markers

## Introduction

Survival of plants under adverse environmental conditions relies on integration of stress adaptive metabolic and structural changes into endogenous developmental programmes. Abiotic environmental factors such as drought and salinity are significant plant stressors with a major impact on plant development and productivity, thus causing serious agricultural yield losses (Muscolo *et al.*, 2011; Flowers and Muscolo, 2015). Plant stress responses are dynamic and involve complex cross-talk between different regulatory levels, including adjustment of metabolism for physiological and morphological adaptation (Saito and Matsuda, 2010). The most well documented changes in plant metabolism under drought or salinity stress are related to the accumulation of certain metabolites, such as proline (Yoshiba *et al.*, 1995; Kesari *et al.*, 2012), soluble carbohydrates, glycine betaine, and γ-aminobutyric acid (GABA) (Renault *et al.*, 2013), that maintain the osmotic compatibility within the cell, decrease the entropy levels, and support the maintenance of proteins in the folded native tertiary structures. The accumulation of other metabolites such as ascorbate and glutathione (Miller *et al.*, 2007; Tunc-Ozdemir *et al.*, 2009; Raschke *et al.*, 2011; Szarka*et al.*, 2012) also contributes to substantial reduction of the harmful effect of reactive oxygen species (ROS) generated by abiotic stresses, while ROS theselves might act as an important messenger during stress responses (Suzuki *et al.*, 2012). Metabolic activities in response to stress occur more quickly than transcriptional responses (Fraire-Velázquez and Balderas-Hernández, 2013). Thus, using metabolomics as a diagnostic tool within the right discovery context provides a powerful means to gain a better understanding of physiological responses to stress. Some metabolic changes are common to different stresses, whereas others are specific. Comparison of the metabolic profiles of lentil accessions with different tolerance to salinity and drought stresses (Muscolo *et al.*, 2007, 2014; Sidari *et al.*, 2008) may show overlap, but also specificity, in metabolic adjustments under different conditions.

The present study addresses components of drought and salinity stress adaptation of four lentil accessions, indicating metabolic adjustments related to the differences in stress tolerance. The accessions Pantelleria (PAN) and Ustica (UST) are native and cultivated in the homonymous small islands close to Sicily (Southern Italy), Castelluccio di Norcia (CAST) is a local population cultivated in the Umbria region (Central Italy), and Eston (EST) is a Canadian commercial variety. Lentil (*Lens culinaris* M.), a source of high quality protein in the human diet, is an important legume in the farming systems of the Mediterranean area where plants are exposed to strict environmental constraints (mainly drought and salinity) limiting their cultivation. In this research, the effects of salinity and drought on two developmental stages of the plants, germination and early growth, were monitored during cultivation in a phytochamber with the aim of demonstrating morphological, physiological, and metabolic traits associated with drought and/or salinity stress tolerance. An automated platform serving as a Complex Stress Diagnostic System was used for computer-controlled watering and digital imaging to record the stress responses of individual plants by monitoring plant growth and physiological properties derived from top and side view images of visible (VIS) and near-infrared (NIR) reflection and fluorescence (FLUOR) emission. Further major objectives of this research were the identification of specific changes in the metabolite profile in response to abiotic stress conditions through gas chromatography–mass spectrometry (GC-MS) analysis.

Understanding the biochemical mechanisms involved in plant drought and salinity stress tolerance is still a major challenge in biology and agriculture to identify at an early stage suitable traits that would help plant breeders in specific selection programmes. Selecting cultivars with diverse physiological responses to different stresses would provide novel options to growers under different environment constraints.

## Materials and methods

### Seed germination

Seeds of each lentil cultivar were germinated in 9cm diameter Petri dishes containing seed germination paper filter (Whatman) moistened with 3ml of distilled water (control), with 18% (w/v) polyethylene glycol (PEG) 6000 solution or 150mM NaCl solution. Seeds were germinated in a growth cabinet at 25 °C with 16h light/8h darkness. Seed germination percentages were recorded daily for up to 72h, using radicle extrusion (2mm) as a criterion. At the end of the treatment phase (72h), the water content was measured and expressed as a percentage. Freshly sampled material of germinating seeds (*n*=8 biological replicates) was harvested at noon, immediately frozen in liquid nitrogen, and stored at –80 °C until use for GC-MS analysis (see below).

### Plant material and stress treatment

The four lentil cultivars were grown under water-sufficient, NaCl, and water-limiting conditions in a controlled climate chamber. A complete block and split-plot design of 10 blocks with 12 plants including each genotype×treatment (4×3) combination was used. Each treatment and cultivar was represented by 10 replicate pots with one plant per pot. Pots were randomized within the blocks. Plants were cultivated in 10cm pots on a 7.5cm deep layer of substrate 2 (Klasmann-Deilmann GmbH, Geeste, Germany). All pots were weighed and adjusted to 70% field capacity. Seedlings were cultivated for 15 d either in unchanged soil (control) or in soil conditioned with 150mM NaCl for salt stress or modified for osmotic stress (–0.88MPa) by using 18% PEG 6000. Moisture levels were kept constant by supplying water using a balance/watering set-up. Seeds were germinated and plants were grown and monitored in the IPK high-throughput plant phenotyping platform (LemnaTec) for small plants, in a phytochamber in 12h days [240 μmol m^−2^ s^−1^ photosynthetically active radiation (PAR)] with 26 °C and 75% relative humidity, and 22 °C and 70% relative humidity at night.

During cultivation, plant morphological parameters (extracted from RGB images, top, side view), bulk fluorescence signals (FLUOR), and near infrared reflection (NIR, related to moisture content) were recorded (Junker *et al.*, 2015). Image analysis was carried out using the IAP software tool (Klukas *et al.*, 2014). The FLUOR signal value was gauged towards the detection of chlorophyll (red fluorescence) and was calculated from the captured colour hue value in the HSB (hue, saturation, brightness) colour space by linear scaling of the range between the extremes of yellow (60 °) and red hue (0 °) to values between 0 and 1. To include furthermore the observed fluorescence intensity, the colour hue-based value was multiplied by the brightness value also linearly scaled to values ranging from 0 (dark) to 1 (maximum detectable brightness). The calculated fluorescence signal value may thus range from 0 to 1, but a cut-off value of ~0.137 was used to separate foreground pixels from the background. NIR reflection values of the plant tissue were extracted from the captured NIR images (detected in the 1400–1510nm wavelength band) by applying the scaled-down and aligned plant image mask derived from the fluorescence images. The detected grey values were linearly scaled to the range of 0 (black) to 1 (white = maximum detectable signal). For easier interpretation of the results, the values were inverted and NIR intensity was defined as 1–observed grey level, so that high values reflect high absorption, related to high water content.

Extracted values of selected traits were subjected to statistical analysis using R [analysis of variance (ANOVA) and subsequent post-hoc analysis by Tukey’s range test]. After 15 d of drought and salinity stresses, plants were harvested 4–6h after the beginning of the light period. For metabolic profiling, whole shoots were harvested and immediately frozen in liquid nitrogen, and stored at –80 °C until used for GC-MS analysis.

### GC-MS

A 15mg aliquot of shock-frozen cotyledon, radicle, or shoot material was ground to homogeneity and extracted as described previously (Riewe *et al.*, 2012). The total number of samples for MP analysis was 288 considering seed, root, and shoot samples (*n*=8 biological replicates). In-line derivatization/gas chromatography coupled to electron impact ionization-time of flight-mass spectrometry (GC/EI-TOF-MS) was performed using a Gerstel MPS-XL auto sampler (Gerstel, Mühlheim, Germany) in combination with an Agilent 7890 gas chromatograph (Agilent, Santa Clara, CA, USA) attached to a Leco Pegasus HT mass spectrometer (LECO, St. Joseph, MI, USA) (Riewe *et al.*, 2012). Analytes with known chemical structure were identified and annotated when matching to a metabolite in the mass spectrum reference library provided by the Golm Metabolite Database (http://gmd.mpimp-golm.mpg.de/) according to both, fragment spectrum and retention index. Quantitative data were extracted using Target Search (Cuadros-Inostroza *et al.*, 2009) and were normalized to abundance of internal standard, sample weight, and, in the case of cotyledon and root samples, also for extraction day/detector response differences. Biological replicate metabolite abundances (*n*=8) above or below the median plus or minus twice the standard deviation were regarded as outliers and excluded from further analysis.

The data were analysed as described in Roessner *et al.* (2001) and are presented as fold changes compared with the reference, which was set to 1. Fold changes <1 were inverted and multiplied by –1 to aid interpretation. Differences between two treatments were considered significant when the *P*-value (calculated using a Student’s *t*-test) was <0.05.

## Results

### Seed germination (*in vitro*)

**Fig. 1.**
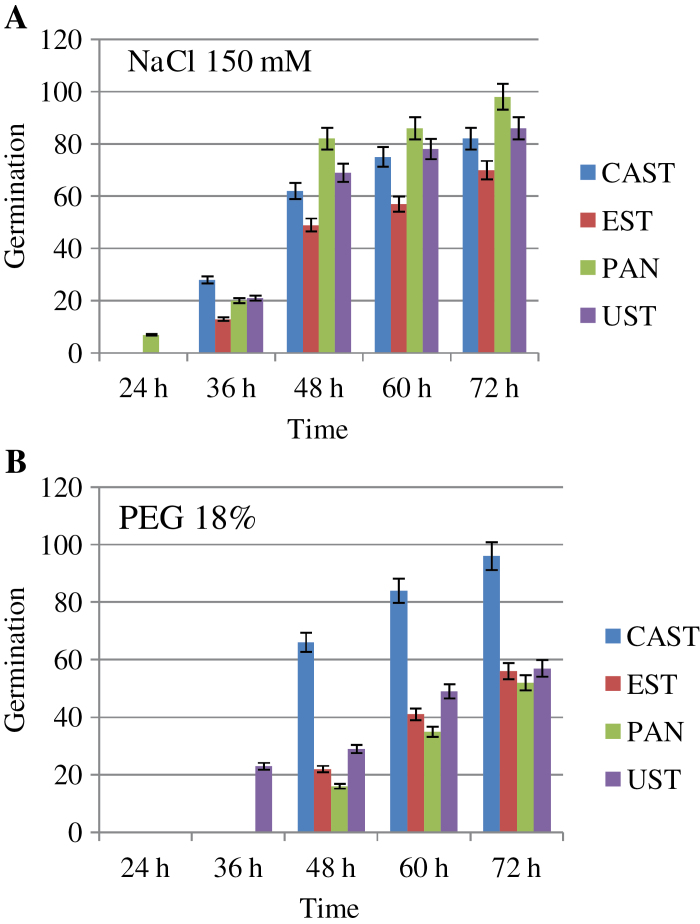
Germination percentage of Castelluccio di Norcia (CAST), Eston (EST) Pantelleria (PAN), and Ustica (UST) genotypes treated with 150mM NaCl (A) or 18% polyethylene glycol (PEG) (B). Germination was detected at 24, 36, 48, 60, and 72h. Data are expressed as a percentage of the control. Values are the mean of eight replicates. Bars are the SEMs. (This figure is available in colour at *JXB* online.)

The results showed a relationship between imposed stresses and performance of the lentil cultivars analysed. The ranking of genotype resistance/susceptibility according to their germination frequencies (Fig. 1) was as follows: NaCl resistant > susceptible, PAN > UST > CAST > EST; PEG resistant > susceptible, CAST > UST > EST > PAN. PAN and UST germinated better in NaCl (98% and 86%) than in PEG (56% and 57%), EST germinated less in the presence of both stresses (70% in NaCl and 56% in PEG), and, conversely, CAST germination was high under both stresses (96% in PEG and 82% in NaCl). The sensitivity of lentil to salinity and PEG was also indicated by root morphology. PAN and UST had longer roots in the presence of salinity; conversely, CAST and EST showed thicker radicles under osmotic stress (data not shown).

### GC-MS analysis

The GC-MS analysis of germinated seeds (cotyledons and radicles) (Tables 1, 2) showed that in the different genotypes, the metabolite profiles had the same trends with respect to the treatments but with significant quantitative differences.

**Table 1.** Main effects of 72h drought (18% PEG) and salinity (150 mM NaCl) stresses on cotyledon metabolite contents in the lentil genotypes Pantelleria (PAN), Ustica (UST), Castelluccio (CAST), and Eston (EST).

Metabolites	PAN		UST		CAST		EST	
	NaCl	PEG	NaCl	PEG	NaCl	PEG	NaCl	PEG
**Polyamines**								
Putrescine	NS	19.6	NS	22.0	NS	12.5	NS	13.1
Cadaverine	NS	18.9	NS	52,8	NS	22.2	7.3	15.9
**Organic acids**								
Acotinic acid	–5.3	–3.3	–4.6	–9.3	–10	NS	–6.9	–6
Ascorbic acid	–3.39	NS	–7.56	NS	NS	NS	–4.33	NS
Butanoic acid	NS	NS	NS	25.9	–4.11	5.25	NS	NS
2-Aminobutanoic acid	–3.31	NS	NS	8.75	NS	NS	NS	4.92
Citric acid	–6.58	–21.5	–12.3	–57.2	–7.05	–16.3	–10.7	–51.6
Erythronic acid	–4.8	–7.7	–6.8	–2.7	–5.5	–2.1	–6.2	–2.0
Fumaric acid	–4.9	NS	–3.6	NS	–4.2	NS	–4.5	NS
2-Oxolgutaric acid	NS	–2.72	NS	NS	–2.92	–1.88	–3.32	–2.91
Glyceric acid	–10.8	–9.6	–6.6	–4.6	–6.8	NS	–16.6	–15.0
3-Phosphoglyceric acid	NS	3.29	NS	2.91	NS	NS	NS	NS
Isocitric acid	1.6	40.1	1.5	16.7	6.7	10.8	2.6	17.4
Malic acid	–9.65	–1.7	–14.7	NS	–13.3	–2.42	–13	NS
Malonic acid	–3.22	1.47	NS	4.59	–5.14	2.02	–3.49	4.24
Nicotinic acid	NS	317	NS	510	NS	182	NS	184
Phosphoric acid	–9.4	–5.2	NS	1.9	–18	4	–6.8	1.4
2-Aminopropanoic acid	NS	6.2	NS	4.3	NS	2.2	NS	2.0
Pyroglutamic acid	NS	NS	–2.1	NS	–2.1	NS	–1.97	NS
Pyruvic acid	–3.3	–2.7	–3.4	–2.9	–3.7	NS	–4.7	–3.8
Succinic acid	–4.0	–4.1	–4.0	–2.1	–6.1	–3.2	–6.5	–4.4
Threonic acid	–16.4	–32.8	–15.3	–6.67	–19	–5.22	–46.8	–19.9
5-Aminovaleric acid	–3.7	NS	–4.1	NS	–3.28	NS	–3.53	NS
**Sugars and polyols**								
Fructose	NS	2.7	NS	7.3	NS	NS	NS	NS
Fructose-6-P	NS	–2.0	NS	–2.1	–4.1	NS	–2.6	NS
Glucose	–5.6	3.8	–5.3	4.0	–6.5	NS	–2.7	NS
Glucose-6-P	–2.9	NS	–2.0	NS	–3.6	NS	–2.1	NS
Glycerol-3-P	NS	3.3	NS	2.9	NS	NS	NS	NS
*myo*-Inositol-1-P	NS	3.8	NS	3.9	–2.7	1.8	NS	1.9
Ribose-5-P	–2.4	NS	NS	–1.6	–3.3	NS	–2.9	–4.3
Ribulose-5-P	NS	NS	–2.2	NS	7.9	NS	–4.4	NS
Xylose	–2	8.1	NS	7.1	NS	7.2	NS	8.1
**Amino acids**								
4-Hydroxyproline	NS	18.6	NS	10	NS	7.0	NS	14.9
β-Alanine	–2.2	2.1	NS	NS	–2.4	NS	–1.85	NS
Alanine	–2.65	NS	–3.2	NS	–3.8	NS	–3.45	NS
Arginine	NS	20	NS	11.5	NS	8.7	NS	5.7
Asparagine	NS	181	NS	86.1	NS	59.5	NS	86.1
Aspartic acid	–8.17	–1.91	–8.17	–1.91	–10	NS	–7.55	NS
Glutamic acid	NS	NS	–2.19	NS	–2.19	NS	–2.02	NS
Glycine	2.9	35	3.3	20.1	3.1	24.6	3.3	21.2
Histidine	NS	149	NS	156	NS	80	NS	69
Homoserine	–4.6	NS	–3.0	NS	–3.4	NS	–7.15	NS
Isoleucine	NS	46.4	NS	79	NS	34	NS	57
l-Dopa	NS	78	NS	15	NS	10	NS	13
Leucine	NS	27	NS	42.4	NS	17.5	NS	34.2
Lysine	1.8	13.1	2	12.4	1.9	NS	3.7	NS
Methionine	NS	54.1	NS	29.3	NS	32.5	NS	34.1
Ornithine	NS	268	NS	287	NS	206	NS	268
Phenylalanine	NS	21.9	NS	58.7	NS	10	NS	38.5
Proline	5.9	89.8	2.4	94.7	32.5	72.5	47.3	85.3
Serine	NS	2.2	NS	3.4	NS	NS	NS	NS
Threonine	NS	11.4	NS	10	NS	10	NS	7.1
Tryptophan	NS	77.3	NS	97	4.95	59.9	4.35	68.1
Tyrosine	NS	10.7	NS	9.35	NS	3.7	NS	3.5
Valine	NS	35.9	NS	57	NS	21.5	NS	54.1
**Others**								
Dihydrosphingosine	–4.5	–6.7	–4.8	–4.8	–3.6	–5.8	–14.3	–7.5
Urea	NS	41.1	NS	51.9	NS	30.7	NS	29.1
Oxalacetate	NS	22.6	NS	28.7	NS	17.8	NS	11

Interestingly, polyamine contents were similar to the control in all genotypes under NaCl, while their contents increased more in UST and PAN than in CAST and EST under PEG. Organic acids significantly decreased compared with the respective controls, except for isocitric, nicotinic, and oxalacetic acids that increased considerably in PEG-treated plants. Regarding sugars and polyols, the greatest qualitative differences observed among the genotypes were related to glycerol-3-P, fructose, and glucose that increased only in PEG-treated PAN and UST. Additionally, the majority of amino acids increased in PEG-treated genotypes, to a greater extent in PAN and UST. Serine was detected only in PEG-treated PAN and UST. Alanine, β-alanine, and homoserine were the only amino acids that decreased under NaCl in all genotypes. Proline was lower in NaCl-treated PAN and UST than in CAST and EST. Urea increased only in PEG-treated genotypes and much more in PAN and UST. Dihydrosphingosine decreased more under PEG than under salinity in PAN, UST, and CAST. An opposite trend was observed for EST. In roots (Table 2), fewer metabolites were detected than in cotyledons; 11 organic acids, three polyols, two polyamines, and three amino acids were absent. Spermidine was the only polyamine detectable in roots; it increased in PAN under PEG and in the other three cultivars under NaCl. The major changes were in sugar and polyol contents. The metabolites affected most by the stresses were in the order: trehalose, which increased in each cultivar under both stresses except for NaCl-treated UST; maltose, which increased in PEG-treated PAN and in NaCl-treated CAST and EST; proline, which was increased in each cultivar in stress conditions except for PEG-treated CAST and NaCl-treated EST; and isoleucine, which was enhanced in NaCl-treated UST, CAST, and EST.

**Table 2.** Main effects of 72h drought (18% PEG) and salinity (150 mM NaCl) stresses on root metabolite contents in the lentil genotypes Pantelleria (PAN), Ustica (UST), Castelluccio (CAST), and Eston (EST).

Metabolites	PAN		UST		CAST		EST	
	NaCl	PEG	NaCl	PEG	NaCl	PEG	NaCl	PEG
**Polyamines**								
Cadaverine	NS	NS	NS	NS	2.31	NS	NS	NS
Spermidine	NS	4.79	3.7	NS	4.58	NS	4.17	NS
**Organic acids**								
Butanoic acid	2.72	NS	NS	NS	NS	NS	NS	2.28
2-Aminobutanoic acid	NS	2.93	NS	NS	NS	NS	NS	NS
Citric acid	NS	1.97	–3.34	–2.38	NS	NS	–1.2	NS
Erythronic acid	NS	NS	NS	NS	NS	–1.93	NS	NS
Glyceric acid	NS	NS	–2	NS	NS	NS	NS	NS
Isocitric acid	NS	NS	–2.46	NS	NS	NS	NS	1.52
Malic acid	NS	NS	–7.45	NS	NS	NS	–4.9	NS
Nicotinic acid	NS	2.55	NS	NS	NS	NS	NS	NS
**Sugars and polyols**								
Cellobiose	–4.8	–1.69	NS	NS	3.0	NS	NS	NS
Galactinol	NS	NS	4.0	NS	NS	NS	3.76	NS
Galactose-*N*-acetyl			–2.8				–1.46	
Glucose	NS	NS	–5.99	NS	NS	NS	NS	NS
Glycerol-3-P	1.75	2.12	NS	NS	2.38	NS	2.22	1.97
Maltose	NS	4.42	NS	NS	4.41	NS	5.12	NS
*myo*-Inositol	NS	NS	–3.45	NS	2.09	NS	1.8	1.42
Raffinose	5.73	7.28	NS	NS	NS	NS	5.73	10.9
α,α-Trehalose	6.88	8.53	NS	6.87	6.51	4.98	4.48	10.1
Xylose	NS	NS	–2.91	NS	NS	NS	NS	NS
**Amino acids**								
Glutamic acid	2.78	5.45	NS	NS	NS	NS	NS	NS
Glycine	NS	–1.95	–2.1	NS	NS	NS	NS	NS
Guanosine	NS	1.95	NS	NS	NS	NS	2.89	1.7
Isoleucine	NS	NS	–3.91	NS	–2.48	NS	–2.35	NS
Leucine	NS	NS	–3.73	NS	NS	NS	–3.34	NS
Proline	4.44	6.23	7.62	3.74	6.44	NS	NS	6.44
Valine	NS	NS	NS	NS	NS	NS	–1.45	NS

### Seedling growth

**Fig. 2.**
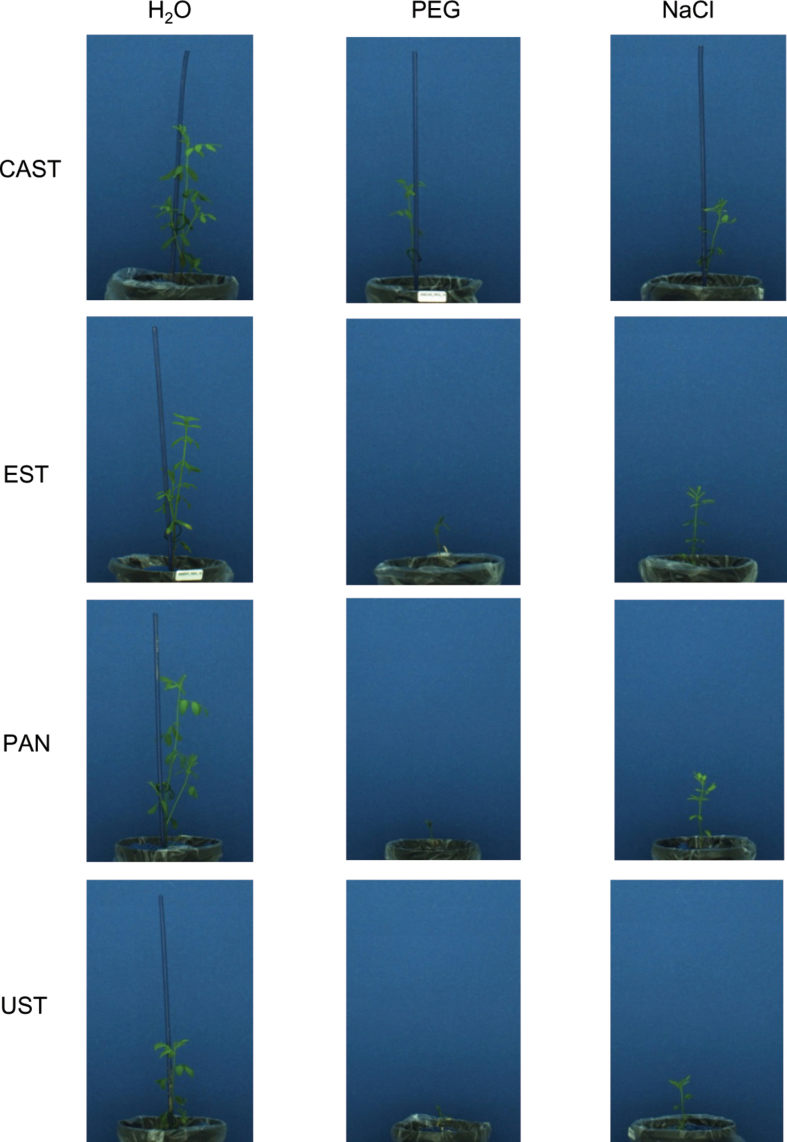
Representative side view RGB images of plants of four lentil accessions at 15 d after sowing under control conditions (H_2_O), osmotic stress (PEG), and salt stress (NaCl).

**Fig. 3.**
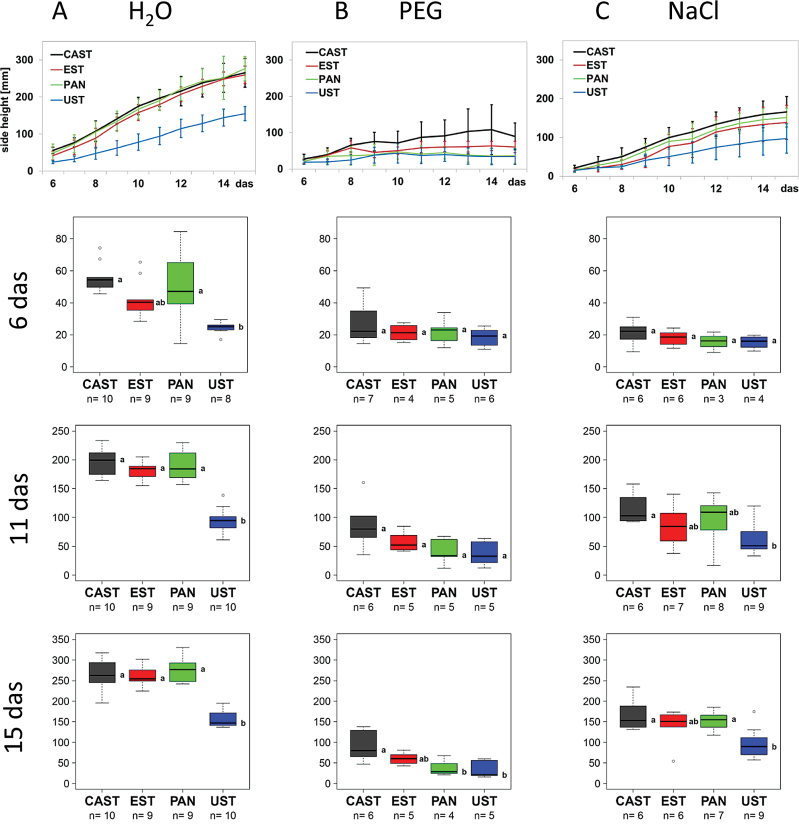
Growth dynamics of four lentil accessions under (A) control (H_2_O), (B) osmotic stress (PEG), and (C) salt stress (NaCl). Growth curves (upper panel, mean values of 4–10 replicates with bars indicating the SEM) represent plant height development as extracted from RGB side images of the plants from day 6 to 15 after sowing (das). Box plots represent variation in the height of plants of the different genotypes at day 6, 11, and 15 after sowing, with the letters indicating significant differences according to Tukey’s test. (This figure is available in colour at *JXB* online.)

**Fig. 4.**
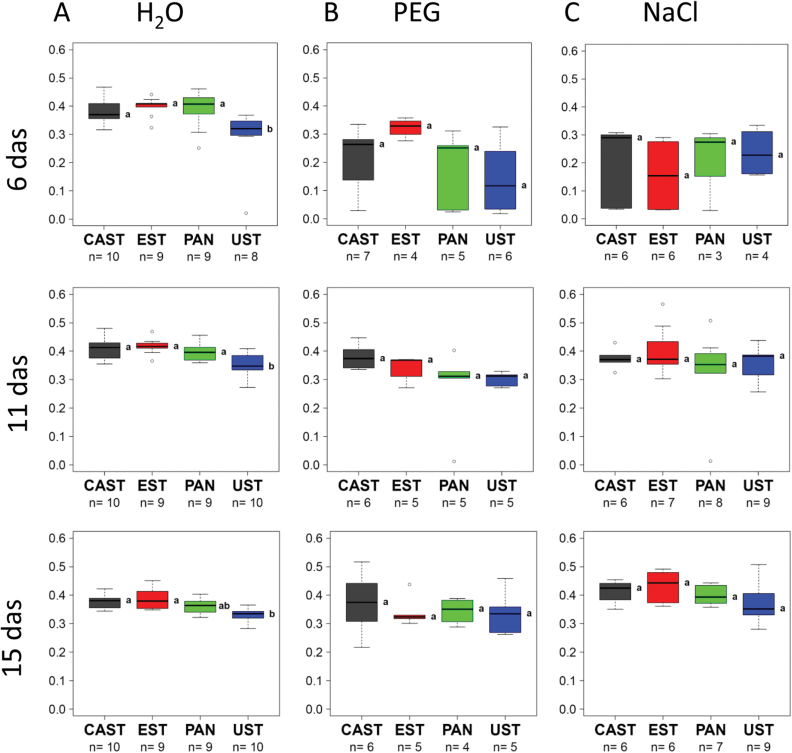
Dynamics of the fluorescence signal of four lentil accessions under (A) control (H_2_O), (B) osmotic stress (PEG), and (C) salt stress (NaCl). Box plots represent variation in fluorescence signal of plants of the different genotypes at day 6, 11, and 15 after sowing (das), with the letters indicating significant differences according to Tukey’s test. (This figure is available in colour at *JXB* online.)

**Fig. 5.**
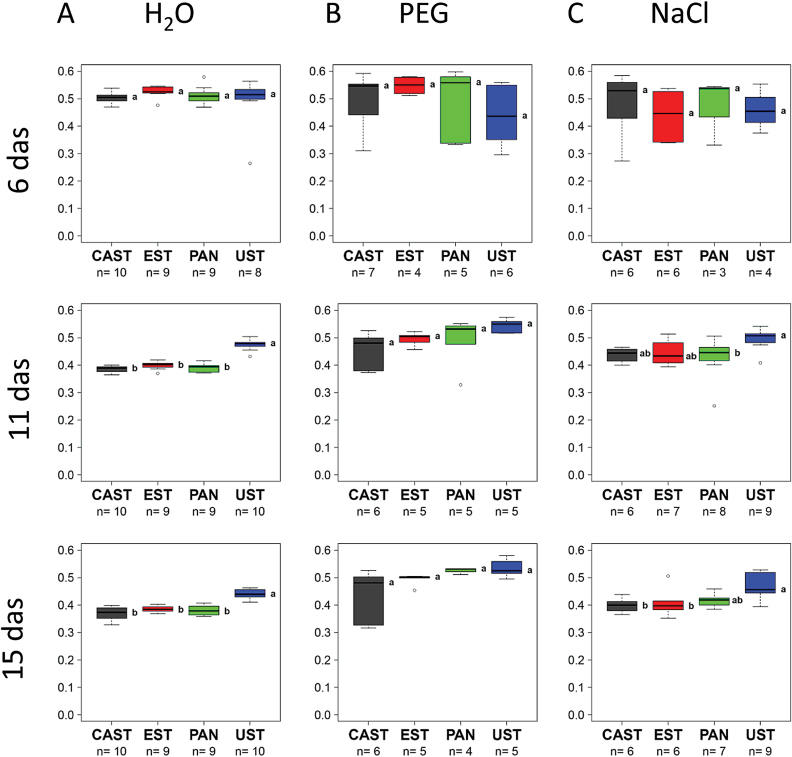
Dynamics of near infrared (NIR) intensity of four lentil accessions under (A) control (H_2_O), (B) osmotic stress (PEG), and (C) salt stress (NaCl). Box plots represent variation in NIR intensity of plants of the different genotypes at day 6, 11, and 15 after sowing (das), with the letters indicating significant differences according to Tukey’s test. (This figure is available in colour at *JXB* online.)

The data on seedling growth were extracted from the images collected from day 6 to day 15 of the cultivation using the IAP software package (Klukas *et al.*, 2014). Under control conditions (Fig. 2, H_2_O; Fig. 3A), UST was smaller and significantly shorter than the three other genotypes, growth of which was very similar over the entire cultivation period (Fig. 3A), and in respect to side view had lower fluorescence intensity (Fig. 4A) and NIR intensity (displayed as 1–observed grey level) (Fig. 5A). Plant growth (monitored as plant height) was severely reduced in all genotypes upon PEG treatment and, except for the earliest time point [6 days after sowing (DAS)], clear differences were apparent between genotypes, ranking CAST > EST > PAN ≈ UST (Figs 2, 3B). Growth depression was less severe for all four genotypes under NaCl than under PEG treatment. UST was smaller than all other genotypes after a few days of treatment (Figs 2, 3C). Little difference was observed among CAST, PAN, and EST that also showed variation over the treatment period (Fig. 3C). Fluorescence signals (Fig. 4) in all accessions were lower in treated than in untreated conditions and varied particularly strongly in the early phases of both stress treatments; however, values increased over time during the treatments, reaching levels similar to the controls at 15 DAS. Also, NIR intensity values were more variable in the stress treatments than in the control (Fig. 5). The pattern of the genotype ranking according to the NIR intensity values (UST > CAST ≈ PAN ≈ EST) was very similar for the NaCl treatment and the control situation (Fig. 5A, C). In the PEG treatment, however, no significant differences were observed among the accessions.

**Fig. 6.**
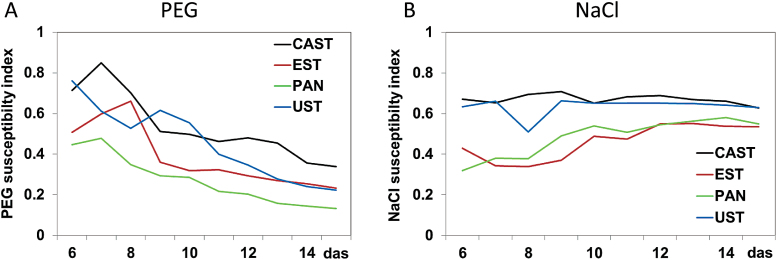
Tolerance indices for four lentil accessions and their growth responses to (A) osmotic stress (PEG) and (B) salt stress (NaCl). Plots represent the ratio of the mean plant height values as extracted from RGB side images per treatment, genotype, and day [6–15 days after sowing (das)] between stress conditions and the control. Tolerance index: 1=full tolerance, 0=total susceptibility. (This figure is available in colour at *JXB* online.)

Using the plant height data, tolerance indices were calculated for all genotypes and every day from 6 DAS to 15 DAS as the ratios of the values in the stress condition/control values (Fig. 6). Tolerance indices dropped over time for all genotypes in the PEG treatment, whereas they were either constantly high (for CAST and UST) or increased moderately from an initial low level (for PAN and EST) upon NaCl exposure. For the PEG treatment, a stress tolerance ranking of CAST > EST ≈ UST > PAN was observed, while a different response was apparent in the NaCl treatment, with a ranking of CAST ≈ UST > PAN ≈ EST.

### GC-MS analysis

The metabolite profile analyses of the shoot material showed interesting differences among the accessions in respect to the stress applied (Table 3). Putrescine increased only in PEG- and NaCl-treated UST, and in NaCl-treated CAST. Spermidine significantly increased in PAN, only under PEG, and in all the other cultivars under both stresses. Cadaverine was enhanced in PEG-treated PAN, in NaCl-treated CAST, and in UST and EST under both stresses. Organic acids were affected differently by the stresses, and the changes were mainly related to the cultivars. Malic, phosphoric, and erythronic acids decreased in PAN under PEG and in the other cultivars under NaCl. The greatest decrease was observed in EST followed by CAST. 2-Aminobutanoic acid increased in PAN mainly under PEG, and in the other three cultivars mostly under NaCl; conversely, butanoic acid decreased (Table 3). Regarding changes in sugars and polyols, raffinose increased mostly in PEG-treated PAN, and in NaCl-treated UST, CAST, and EST. α,α-Trehalose increased in each cultivar under the stresses, except for NaCl-treated EST. Galactose-*N*-acetyl decreased in PEG-treated PAN and in NaCl-treated CAST and EST. The same trend was observed for glucose, with the only exception being the decrease observed in UST under NaCl and in EST under PEG. Erythritol decreased only in NaCl-treated UST, CAST, and EST. Xylose was lower than controls in PEG-treated PAN and in NaCl-treated CAST and EST. The amino acids proline, tryptophan, glycine, leucine, isoleucine, asparagine, valine, and phenylalanine decreased mainly in PEG-treated PAN and in NaCl-treated UST, CAST and EST. Isoleucine, isocitric acid, guanosine, and maltose were not detected in the shoot samples.

**Table 3.** Main effects of 15 d of drought (18% PEG) and salinity (150mM NaCl) stresses on leaf metabolite contents in the lentil genotypes Pantelleria (PAN), Ustica (UST), Castelluccio (CAST), and Eston (EST).

Metabolites	PAN		UST		CAST		EST	
	NaCl	PEG	NaCl	PEG	NaCl	PEG	NaCl	PEG
**Polyamines**								
Cadaverine	NS	4.33	11.5	2.85	13.5	NS	11.5	2.95
Putrescine	NS	NS	1.95	2.18	2.68	NS	NS	NS
Spermidine	NS	5.1	3.17	2.52	4.79	2.52	4.79	3.32
**Organic acids**								
Ascorbic acid	NS	2.61	NS	NS	NS	NS	NS	NS
Butanoic acid	–2.15	–4.5	–4.64	–1.3	–3.89	–1.42	–6.81	–2.28
2-Aminobutanoic acid	2.76	5.58	9.7	3.2	5.41	2.93	4.5	3.64
2-Aminobutanoic acid	1.35	1.35	NS	NS	NS	NS	NS	NS
Erythronic acid	NS	–1.68	–1.55	NS	–1.98	NS	–2.61	NS
Galacturonic acid-1-P	–2.12	–6.55	–5.64	–2.02	–6.86	NS	–7.20	NS
2-Oxoglutaric acid	NS	NS	–1.87	NS	–2.3	NS	–2.95	NS
3-Phosphoglyceric acid	NS	NS	NS	NS	1.85	1.42	1.75	1.93
Itaconic acid	NS	1.97		NS	NS	NS	2.38	NS
Malic acid	NS	–2,97	–2.85	NS	–5.8	NS	–6.03	NS
Malonic acid	NS	NS	–1.98	NS	–2.12	NS	–2.47	NS
Nicotinic acid	NS	NS	NS	1.78	1.65	NS	NS	NS
Pyroglutamic acid	NS	NS	NS	NS	NS	NS	–1.33	NS
Threonic acid	2.35	0	1.75	NS	1.55	NS	0	2.72
5-Aminovaleric acid	NS	–2.81	NS	NS	–5.37	NS	–3.45	–1.55
**Sugars and polyols**								
Cellobiose	NS	1.87	10.1	NS	5,34	NS	NS	NS
Erythritol	NS	NS	–2.53	NS	–1.98	NS	–2.59	–1.38
Galactinol	NS	NS	NS	NS	3.2	NS	NS	NS
Galactose-*N*-acetyl	NS	–3.11	NS	NS	–3.63	NS	–3.96	NS
Glucose	NS	–11.74	–11.67	NS	–11.66	NS	–12.15	–3.41
Glycerol-3-P	NS	NS	NS	NS	1.45	NS	NS	1.3
*myo*-Inositol	–1.34	–1.55	NS	NS	NS	NS	–1.0	NS
*myo*-Inositol-1-P	NS	NS	–2.35	NS	–2.1	NS	–2.62	NS
Raffinose	3.29	10.1	6.72	NS	8.54	NS	2.59	NS
α,α-Trehalose	2.76	3.81	7.64	7.25	4.98	2.76	NS	4.24
Xylose	NS	–2.43	NS	NS	–2.91	NS	–2.96	NS
**Amino acids**								
Asparagine	NS	–1.55	–1.3	NS	NS	NS	–1.35	NS
Aspartic	1.84	NS	1.66	NS	1.78	NS	NS	NS
Glutamic acid	NS	NS	NS	NS	NS	NS	–1.37	NS
Glycine	NS	NS	NS	NS	–1.72	–1.57	–1.35	–1.95
Isoleucine	NS	–3.11	–1.57	NS	–2.41	–1.79	–2.62	–2.82
Leucine	–2.1	–2.83	–1.93	NS	–2.54	–1.82	–2.76	–2.34
Phenylalanine	NS	–2.44	–1.36	–1.52	–1.85	–1.7	–2.13	–2.32
Proline	NS	NS	3.2	1.72	1.84	NS	NS	NS
Tryptophan	NS	–3.55	–1.97	NS	–2.6	–1.48	–2.46	–1.91
Valine	NS	–1.96	NS	NS	–1.73	NS	NS	NS
Phosphoric acid	NS	–2.08	–2.57	NS	–3.23	NS	–3.23	NS

## Discussion

Different genotypes differ in growth rates and productivity. The physiological or genetic mechanisms that underlie such natural variation are largely untapped resources that not only may provide valuable information on the capacity and performance of different cultivars under different environmental conditions, but also are an invaluable genetic resource that can be used to improve yield (Flood *et al.*, 2011; Lawson *et al.*, 2012). Knowledge of this natural diversity will encourage the use of new cultivars (with desirable traits) in a bid to improve crop yields. The present results demonstrated natural diversity among the cultivars studied, showing differential growth properties of the four accessions in control conditions. CAST, whose height was similar to that of PAN and EST in unstressed conditions, grew better than the other genotypes under both stresses, showing a greater tolerance to the osmotic stress. When compared with their performances under control conditions, UST was tolerant to salt stress but intermediately tolerant to drought, while EST was sensitive to salinity but showed average tolerance to osmotic stress, confirming previous findings of Sidari *et al.* (2008) and Muscolo *et al.* (2014). PAN appeared to be the most sensitive to osmotic stress, and less tolerant to salinity than CAST and UST. In stressed vegetation, leaf chlorophyll content decreases, thereby changing the proportion of light-absorbing pigments, leading to a reduction in the overall absorption of light. These changes affect the spectral reflectance signatures of plants, with relative differences in green, red, and blue reflections. Detecting deviations from the normal (unstressed) spectral reflectance patterns is the key to interpreting plant stress. Thus, fluorescence signals, related to the leaf chlorophyll content, can be good indicators of crop health status (Long *et al.*, 2006; Wu *et al.*, 2008; Zhu *et al.*, 2010; Parry *et al.*, 2011; Raines, 2011). Fluorescence signals were lower in treated plants than in the controls, suggesting that drought and salinity may exert negative effects on chlorophyll contents and thus on the photosynthetic activity of seedlings. In seedlings under drought conditions, and to a minor extent in salt-susceptible seedlings, a decrease in NIR intensity (related to leaf water content) was observed. Given that photosynthesis is clearly compromised by moisture stresses, it would not be surprising that under drought and salinity stress conditions, the leaves of tolerant accessions retain more chlorophyll than those of less tolerant ones. These observations warrant further detailed analyses as retention of chlorophyll can be expected to support a higher rate of photosynthesis, so that less transpiration would be required to generate a given quantity of assimilate (Flexas *et al.*, 2011). A further observation of common responses to both stresses was a shift of carbohydrate production from glucose to raffinose and trehalose, which are recognized as important metabolites in the dessication tolerance of plants (Ahmad and Rasool, 2014). On the other hand, the data revealed differences between the germination and seedling growth stage in the ranking of accession resistance/susceptibility to the different stresses, differences in the stress tolerance of the diverse accessions, and differences in the metabolite levels between cotyledon and shoot tissue of the same genotypes subjected to the same stress. Stress tolerance may thus be considered as a developmentally regulated, stage-specific phenomenon such that tolerance at one stage of plant development may not be linked to tolerance at other developmental stages, as already reported by Sadat Noori and McNeilly (2000). The results also showed that salinity and drought affected metabolite concentrations differently in a genotype-dependent manner, as also demonstrated by Genga *et al.* (2011). A widely discussed theory of the main mechanisms by which plants cope with water deficits or salinity is the maintenance of a positive cell turgor by osmotic adjustment achieved via the active accumulation of compatible solutes (Morais *et al.*, 2012*a*, *b*). However, many previous analyses of metabolic responses of plants to drought or salinity stress were limited to the analysis of one or two classes of compounds considered as ‘role players’ in the development of tolerance. This approach may have biased the observation of metabolic differences induced by the different stresses. In contrast, the application of a non-targeted metabolomic approach provides a wider perspective of metabolic responses to stress and supports the discovery of novel and stress tolerance-specific metabolic phenotypes (Setia and Setia 2008; Weckwerth and Kahl, 2013). The GC-MS-based metabolite profiling of cotyledons of different lentil accessions performed here showed that drought and salinity stress altered a larger number of metabolites than previously reported. The changes differed with respect to the stress applied and to the accession analysed. Furthermore, the magnitude of metabolic alterations in response to stress correlated with the sensitivity/tolerance phenotype observed: drought affected ~30–40% of the measured metabolites in cotyledons of UST and PAN (drought-sensitive accessions) compared with 10–15% in CAST and EST (drought-tolerant cultivars). Similarly, substantial differences in the metabolic responses were observed when salt-sensitive (EST and UST) and salt-tolerant (CAST and PAN) accessions were compared. Interestingly, the glycolytic pathway was affected by both stresses, monitored as lower amounts of pyruvic acid, the product of glucose catabolism. Drought and salinity stresses also affected the tricarboxylic acid (TCA) cycle, as shown by the decrease in citric, aconitic, 2-oxoglutaric, and succinic acids. An increase in methionine, isoleucine, valine, arginine, proline, and histidine in all genotypes, and in phenylalanine, tyrosine, ornithine, and asparagine levels mainly in the PEG-sensitive accession was detected. These data are in agreement with those of Witt *et al.* (2012), who showed increased contents of some amino acids, including proline, tryptophan, phenylalanine, and histidine, in maize hybrids subjected to drought stress. In agreement with Kalamaki *et al.* (2009), it was also shown here that ornithine accumulation in higher plants was coupled with the production of a pool of osmoprotectants that contributed to the improvement of stress tolerance.

In previous studies, amino acid accumulation in plants exposed to abiotic stress (Kaplan and Guy, 2004; Brosche *et al.*, 2005; Zuther *et al.*, 2007; Kempa *et al.*, 2008; Sanchez *et al.*, 2008*a*, *b*; Usadel *et al.*, 2008; Lugan *et al.*, 2010) was mainly attributed to an enhanced stress-induced protein breakdown as a result of cell damage (Widodo *et al.*, 2009) and not to a compensatory processes as was assumed. The increase in polyamines, sugars, and polyols under PEG suggested a central role for these compounds as regulators of gene expression and signal molecules, as already demonstrated by Hoekstra *et al.* (2001), Gill and Tuteja (2010), and Merchant and Richter (2011).

Apart from the accumulation of proline, in NaCl-treated CAST and EST, no accumulation of the other traditional osmoprotectants, such as amino acids, soluble sugars (glucose and xylose), *myo*-inositol, and the most common organic acids, was detected in the cotyledons of all genotypes during salinity stress. This suggests that salinity and drought stresses, both considered as inducers of osmotic stress, activate different biochemical pathways to enhance tolerance in seeds during germination, suggesting that salinity may affect the germination process of the sensitive accessions much more for ionic toxicity than osmotic potential.

Comparing the most drought-sensitive genotype (PAN) with the most drought-resistant one (CAST), substantial differences were highlighted in the metabolic responses to osmotic stress. In particular, amino acids and sugars significantly increased in the sensitive genotype, while organic acids decreased, suggesting a metabolic flexibility of the sensitive genotype, shifting the biochemical products from growth to survival, producing the osmolytes necessary to contrast the external osmotic potential. This hypothesis is supported by the greatest increase in l-DOPA in the sensitive genotype. l-DOPA has antioxidant properties, protecting cellular structures from ROS in stress conditions, as already demonstrated by Hachinohe *et al.* (2004) and Soares *et al.* (2014). Additionally, the observed increases in ornithine and arginine, mainly in the PEG-sensitive accessions (PAN and UST) may be explained by their roles as osmoprotectants and precursors of polyamine synthesis (also increased) that are known to participate actively in plant protection from osmotic stress (Kalamaki *et al.*, 2009).

All these data show a general down-regulation of energy-consuming processes, demonstrating a large-scale reprogramming of metabolism under drought stress conditions. Under salinity conditions, the observed decrease in homoserine and alanine contents suggests an imbalance in sulphur and ammonium assimilation, respectively, potentially due to a lack of available energy (Kopriva *et al.*, 2002; Khan *et al.*, 2008) in roots.

Comparing the most salt-sensitive genotype (EST) with the most tolerant one (PAN), in the former an accumulation was found of proline, an osmoprotectant produced by stressed plants as a primary defence response to maintain the osmotic pressure in a cell (Nanjo *et al.*, 1999; Roosens *et al.*, 2002; Qu *et al.*, 2005; Funck *et al.*, 2008). The novel and most important differences found in this study between the most NaCl-tolerant genotype (PAN) and the most sensitive one (EST) were related to the decrease in threonic acid levels. Under stress conditions, threonic acid is oxidized to threarate (Parsons *et al.* 2011), a biochemically compatible compound (Guerrier *et al.*, 2000; Jouve *et al.*, 2004) that increases in concentration to increase cellular osmolarity. The decrease in threonic acid in the more stressed genotypes confirms the previous hypothesis that under stress the metabolic pathway is shifted to osmolyte production.

Highly significant changes were also observed in roots, the first plant organ to be negatively impacted by soil water deficit, and usually used to investigate diverse responses to dehydration. A major quantitative trait locus (QTL) has been detected that accounts for 33% of the variation in root biomass, which is one of the principal factors that confer a drought tolerance advantage to plants, among many other constructive mechanism responses to water deficit (Gaur *et al.*, 2008). The greater increase in phenylalanine and tyrosine in the two drought-sensitive accessions may be related to the initiation of lignification, a mechanism to alleviate damage from drought stress at the root level, as reported by Terzi *et al.* (2013) in *Ctenanthe setosa*.

Under salinity, a decrease in the amounts of acids of the TCA cycle and in amino acid levels, except for proline that was high, was found in UST roots. An altered TCA cycle is an expected response for plants experiencing stress that is common for a number of species under investigation, for example grapevine (Cramer *et al.*, 2007), the halophyte *Limonium latifolium* (Gagneul *et al.*, 2007), *Arabidopsis* (Gong *et al.*, 2005; Sanchez *et al.*, 2008*b*), *Lotus japonicus* (Sanchez *et al.*, 2008*a*), and rice (Zuther *et al.*, 2007). The present results suggest that the differences in stress tolerance of the different genotypes at this stage can be attributed to a reduction in TCA cycle activity resulting in impaired energy metabolism, with consequences on the ability of seedlings to acquire water and to support transport processes.

Different stress tolerance rankings (according to growth) and metabolite profiles were detected in seedlings as compared with germinating seeds. In particular, the UST genotype, that ranked second in the resistance to NaCl and PEG upon seed germination, was the least tolerant to both stresses at the seedling stage. The changes in stress tolerance of the different accessions may be associated with differences detected in shoot trehalose content. Trehalose levels are generally quite low in plants and, as demonstrated by Goddijn and van Dun (1999) in *Selaginella lepidophylla* under drought conditions, they increased under stress conditions to protect proteins and membrane structures. The present data agree with these results, showing that the genotypes more susceptible to abiotic stress accumulated more trehalose than the others, potentially due to a greater need to protect their cellular structures from osmotic damage. In leaves, no other significant changes were observed that could explain the differences in the stress tolerance of the analysed accessions.

In summary, the integrated evaluation of the metabolomic results and the phenotypic data revealed that environmental adaptation is under tight regulation, which is critical for plant survival. Many components of this regulatory network are involved in responses to different stresses but may function antagonistically or some responses are prioritized over others, compromising plant resistance to multiple stresses simultaneously.

## Conclusion

Natural stress tolerance is a very complex phenomenon involving numerous metabolites and metabolic pathways. Analyses of metabolic adjustments of genotypes with different levels of stress tolerance provide important complementing evidence for better understanding of the role of different metabolites in the acclimation to harsh environments. The results of the present study suggest that the metabolic adjustments in response to the adverse conditions are transient and depend on the type and severity of the stress, showing also that the stress tolerance at the seed stage does not ensure the establishment and growth of the seedling under stress conditions. A genotype tolerant to salinity or drought at the seed stage may invert its degree of resistance, becoming more sensitive with the persistence of the stresses, or more tolerant, developing an adaptative stress system over time. The developmental stage of plants influences the metabolic adjustment with respect to the type of stress. Apart from the well known metabolites, generally tested as markers of abiotic stresses, stress-specific metabolites were identified, in particular ornithine and asparagine as markers of drought stress and alanine and homoserine as markers of salinity stress.
